# Socio-demographic and behavioural determinants of HIV prevalence among homosexual and bisexual men having sex with men (MSM) in India: Integrated bio-behavioural surveillance

**DOI:** 10.4314/ahs.v23i2.8

**Published:** 2023-06

**Authors:** Aridoss Santhakumar, Joseph K David, Jaganathasamy Nagaraj, Malathi Mathiyazhakan, Balasubramanian Ganesh, Natesan Manikandan, VM Padmapriya, Pradeep Kumar, Shobini Rajan, Arumugam Elangovan

**Affiliations:** 1 ICMR-National Institute of Epidemiology, R-127, 2^nd^ Main Road, TNHB, Ayapakkam, Chennai – 600 077, Tamil Nadu, India; 2 Scientist-D (Former), ICMR - National Institute of Epidemiology, R-127, 2^nd^ Main Road, TNHB, Ayapakkam, Chennai – 600 077, Tamil Nadu; 3 National AIDS Control Organization, Ministry of Health Family Welfare, Government of India, 36 Janpath road, New Delhi-110 001, India

**Keywords:** HIV, Men who have sex with men, homosexual, bisexual, India

## Abstract

**Background:**

HIV prevalence among men who have sex with men (MSM) is 16.5 times higher than adult HIV prevalence. With a socio-cultural context that demands marriage, a considerable proportion of MSM in India have female sexual partners and act as a bridge population. Stratified analysis of HIV risk factors among homosexual and bisexual MSM will be instrumental in identifying the high-risk MSM. We aim to identify the socio-demographic and behavioural factors associated with HIV risk among homosexual and bisexual MSM.

**Methods:**

Overall, 23081 MSM were enrolled in the IBBS conducted across India between October 2014 and November 2015. Data and blood samples were collected. Chi-square test, univariate and multivariable logistic regression methods were used in data analysis.

**Results:**

HIV prevalence was significantly higher among homosexual MSM than bisexual MSM. Older age, lesser education, being a sex worker, being married, living with a male or hijra partner, younger age at initiation of MSM behaviour, duration, injecting drugs, and having STI symptoms were associated with higher prevalence. The prevalence of new homosexual MSM was 11.4%. Nearly 75% of the bisexual MSM reported inconsistent condom usage with female partners.

**Conclusion:**

Interventions for early identification of new MSM and advocacy for safe sex with alternative preventive techniques are recommended.

## Introduction

The integrated bio-behavioural surveillance was conducted among HIV high-risk groups (HRGs) in 2014-15 in India to generate evidence of risk behaviours among HRGs. Men who have sex with men (MSM) are key HRGs, as unprotected anal sex is associated with high HIV risk.^1^ The estimated MSM population in India is 3.1 million, of which 0.13 million are estimated to be living with HIV, accounting for 6.3 % of the total PLHIV.^2^ Based on the IBBS data, the HIV prevalence among MSM in 2019 was 4.3%, 16.5 times higher than the adult HIV prevalence (0.26%) in India.^3^

The National AIDS Control program implemented Targeted Interventions (TI) to deliver HIV preventive and management services to HRGs.^4^ With TIs, the overall HIV prevalence among MSM has declined, with considerable heterogeneity in regions of high prevalence and increasing incidence in low prevalence states.^5^ HIV prevention services under TI are focused on high-risk MSM who by definition are ‘primarily self-identified, practicing receptive anal sex and having multiple sexual partners.’ However, MSM practicing insertive or both insertive and receptive anal sex are most likely to be bisexual MSM.^6^ They are considered a substantial bridge population, and their role in disease transmission to the general population is widely discussed. Most married or bisexual MSM are perceived to be heterosexual men and therefore are less likely to be benefited from the targeted interventions.^7^ A qualitative study among bisexual men in Mumbai, India, indicated a lack of bisexual community spaces, expression of compartmentalized private and public identities subject to social and cultural contexts, and a gap in interventions for bisexual men.^8^ Previous literature suggests that MSM report higher rates of mental health issues and resort to substance abuse and unsafe sexual behaviours as coping mechanisms that are often associated with higher HIV prevalence.^9^,^10^ Globally, studies have reported a lower HIV prevalence among bisexual MSM than homosexual MSM but indicated low HIV testing rates and higher rates of inconsistent condom usage with female partners among bisexual MSM.^11^,^12^

Based on the evidence, researchers strongly recommend disaggregated surveillance and data reporting among the subpopulations to enhance the effectiveness of targeted interventions. No studies comparing the prevalence and risk behaviours between homosexual and bisexual MSM in India have been reported. Hence, it becomes essential to identify the behavioural patterns among bisexual and homosexual MSM for better quantification of HIV burden and a deeper understanding of the risk factors between groups. A stratified analysis of the IBBS data will augment the optimization of interventions. Therefore, this study aims to analyse and compare important socio-demographic and behavioural determinants of HIV prevalence among homosexual and bisexual MSM in India.

## Methods

Data was collected from MSM during the integrated Bio-Behavioural Survey conducted in 2014-2015. Male, aged 15 years or more, who had anal or oral sex with a male/ hijra partner in the previous month were included. The sample size was calculated to be 400 at each site. In total, 23,081 MSM were included from 61 randomly selected study sites covering 95 districts in 24 states and union territories across India. The achieved sample size was however lesser than 400 at certain domains because of the non-availability of MSM or a higher refusal rate than expected due to stigma or reluctance to identify themselves as MSM. Study sites were continuous geographical units for which the bio-behavioural estimates were generated. IBBS was conducted for any three months in each site, between October 2014 and November 2015. Recruitment was done by probability-based sampling method with a cluster sampling approach. Two types of cluster sampling approaches; conventional cluster sampling (CCS) and time location cluster sampling (TLCS) were adopted. CCS was used to recruit MSM from conventional clusters such as homes or establishments, whereas TLCS was employed for mobile and dynamic MSM. Data and blood samples were collected from all consenting MSM. A computer-assisted personal interview using a structured questionnaire was conducted by trained personnel for data collection. Samples were collected using the dried blood spot (DBS) method and tested at designated laboratories using the standard two-test protocol. All SOPs were followed as per the operational guidelines as discussed elsewhere.^3^

### Measures

HIV Prevalence: MSM who answered ‘yes’ to the question ‘ever had sex with females’ were grouped as bisexual MSM, and the rest were grouped as homosexual MSM (gay). HIV prevalence was determined for both groups, and within each group, the prevalence was calculated for categorical variables.

Socio-demographic characteristics included information about current age (in completed years), education status, marital status (e.g., single, unmarried, married, separated, and divorced), living with (alone, family/relatives without a sexual partner, female partner, male/hijra partner, and friends/others), age of first sexual intercourse, and duration of MSM activity.

Behavioural Characteristics included information about self-perceived sexual orientation (kothi, panthi, double-decker or others), commercial sexual behaviour, type of sexual partners, consistent condom usage, injecting drug use, alcohol consumption before or during sex, presence of STI symptoms, having heard of HIV and ever tested for HIV.

### Statistical Analysis

Data were summarized using descriptive analyses and tabulated as a weighted representation. The socio-demographic and behavioural factors associated with the risk of HIV infection between the two groups of MSM were analysed by the Chi-square test and compared using risk ratio (RR) with a 95% confidence interval. Odds ratio (OR) with 95% confidence intervals (CI) were calculated using a univariate logistic regression model. For each variable, the category that was normal or assumed to have the least association with HIV risk was taken as ‘reference.’ The adjusted odds ratio (aOR) of the risk factors associated with HIV infection was determined by multivariable analysis. Variables that were marginally significant with P<0.10 in univariate analysis were selected for multivariable analysis. All statistical analyses were done using IBM SPSS software, version 26.0.

## Results

Overall, data collected from 23081 MSM were analysed, of which 11951 (51.8%) were homosexual and 48.2 % (11130) bisexual MSM. About 68.1% were aged 25 and above, 88.4% were literates, 64.1% were never married, and 71.5% were employed (Table S1). Table 1 describes and compares the distribution and HIV prevalence among homosexual and bisexual MSM based on their socio-demographic and behavioural variables. The median age of homosexual MSM was 25 years old (IQR: 8), and that of bisexual MSM was 30 years old (IQR: 11). About 90% of the homosexual MSM were unmarried, while nearly half the bisexual MSM were currently married. Likewise, 66.6% of homosexual MSM identified themselves as kothis while 25% of bisexual MSM identified themselves as panthi. HIV prevalence was significantly higher among homosexual MSM than bisexual MSM (5.2 Vs. 3.6 P<0.001).

**Table S1 (supplementary data file):** Distribution and HIV prevalence by Socio-demographic and behavioural characteristics of MSM in India

Characteristics (N = 23081)	n (%)	HIV (%)
**Age Group (Yrs.)**		
15-24	7364 (31.9)	3.2
> 25	15717 (68.1)	5.0
**Education**		
Literate (Can read and write)	20358 (88.4)	3.9
Illiterate	2705 (11.6)	8.4
**Source of Income**		
Unemployed	2464 (10.8)	5
Student	2653 (11.5)	3
Labourer	7881 (33.9)	4.8
Domestic Servant	536 (2.3)	5.7
Transport worker	600 (2.6)	1.9
Hotel Staff	1505 (6.6)	3.9
Sex work/Masseur	1002 (4.4)	9.4
Others	6405 (27.9)	3.6
**Marital status**		
Never Married	14780 (64.1)	4.7
Currently Married	7132 (30.9)	4.3
Separated/Widowed/Divorced/Others	1144 (5)	2.2
**Currently living**		
Living alone	3641 (15.8)	3.1
Living with family/relatives without sexual partners	12820 (55.4)	4.8
Female partner	4588 (19.9)	3.9
Male/Hijira partner	533 (2.3)	6.5
Living with friends/others	1474 (6.4)	4.5
**Traveled Outside the District**		
No	10844 (47.5)	4.4
yes	12004 (52.5)	4.5
**Age of first sexual intercourse with male/Hijira (In years)**		
<18	14063 (69.3)	1.7
19-24	5247 (25.8)	4.9
>=25	995 (4.9)	4.3
**Duration of MSM behavior (In years)**		
0-1	658 (3.2)	6.2
>1 - 5	3843 (18.9)	1.2
5-10	6120 (30.2)	4.2
>10	9675 (47.7)	6.1
**Self-Identification**		
Bisexual	1431 (6.2)	2.1
Predominantly Kothi (Anal-receptive)	11844 (51.2)	5
Predominantly Panthi (Anal-Insertive)	4261 (18.6)	3.4
AC/DC or Double Decker	5534 (23.9)	4.5
**Commercial Sex Behaviour**		
No	9729 (42.2)	4.9
Yes	13347 (57.8)	4.1
**Alcohol consumption before or during sex**		
No	5193 (43.8)	4.4
Yes	6667 (56.2)	4.1
**Injecting drugs in last 12 months**		
No	22505 (97.5)	4.4
Yes	576 (2.5)	5.7
**Have at least one STI symptom**		
No	18230 (79.1)	4.2
Yes	4819 (20.9)	5.5
**Heard of HIV/AIDS**		
No	1050 (4.6)	3.1
Yes	22017 (95.4)	4.5
**Ever tested for HIV**		
No	4799 (21.8)	2.4
Yes	17195 (78.2)	5.1

**Table 1:** HIV prevalence and relative risk based on socio-demographic and sexual behaviors among Homosexual and Bisexual MSM in India

Variables	Homosexual MSM		Bisexual MSM		RR@ (95% CI)	*P* value
	n	HIV (%)	n	HIV (%)		
	11951	5.2	11130	3.6	1.43 (1.27 - 1.62)	<0.001**
**Age Group (Yrs.)**						
15-24	4866	3.7	2498	2.3	1.66 (1.24 - 2.23)	0.001**
25-34	5395	5.3	4942	3.0	1.77 (1.46 - 2.15)	<0.001**
≥ 35	1691	8.8	3689	5.3	1.65 (1.35 - 2.03)	<0.001**
**Education**						
Literate	10664	4.4	9694	3.4	1.29 (1.12 - 1.48)	<0.001**
Illiterate	1277	12.1	1428	5.2	2.34 (1.79 - 3.06)	<0.001**
**Source of Income**						
Unemployed/Student	3361	4.0	1756	3.9	1.03 (0.78 - 1.37)	0.800
Labourer	3558	5.7	4323	4.1	1.37 (1.13 - 1.67)	0.001**
Domestic Servant	213	7.0	323	4.9	1.43 (0.72 - 2.83)	0.311
Transport worker	198	1.6	401	2.1	0.76 (0.21 - 2.73)	0.681
Hotel Staff	780	6.0	724	1.8	3.36 (1.83 - 6.17)	<0.001**
Sex work/Masseur	687	9.9	316	8.3	1.20 (0.78 - 1.84)	0.403
Others^	3138	4.4	3267	2.8	1.56 (1.20 - 2.02)	<0.001**
**Marital status**						
Never Married	10770	5.2	4010	3.3	1.56 (1.29 - 1.88)	<0.001**
Currently Married	760	6.2	6371	4.1	1.52 (1.12 - 2.06)	0.006*
Separated/Widowed/Divorced/ Others	411	3.6	733	1.5	2.49 (1.15 - 5.40)	0.019*
**Currently living with**						
Living alone	2294	3.5	1347	2.4	1.48 (0.99 - 2.22)	0.053
Family/relatives without sexual	7926	5.5	4894	3.7	1.47 (1.24 - 1.74)	
partner						<0.001**
Female partner	392	4.7	4196	3.8	1.23 (0.77 - 1.97)	0.445
Male/Hijra partner	352	7.2	181	5.0	1.43 (0.69 - 2.98)	0.341
Living with friends/others	966	5.0	508	3.6	1.38 (0.81 - 2.34)	0.208
**Traveled Outside the District (in last 12 months)**						
Yes	5681	5.7	6323	3.4	1.68 (1.42 - 2.00)	<0.001**
NO	6129	4.8	4715	4.0	1.19 (1.00 - 1.43)	0.056
**Age of first sexual intercourse with male/hijra (In years)**						
≤18	8315	5.4	5747	4.3	1.24 (1.07 - 1.45)	0.005*
19-24	2019	5.8	3228	3.3	1.74 (1.34 - 2.24)	<0.001**
≥25	254	3.3	741	1.1	2.89 (1.12 - 7.49)	0.024*
**Duration of MSM behavior (In years)**						
0-1 Yrs	329	11.4	329	0.9	12.8 (3.94 - 41.53)	<0.001**
>1 - 5 yrs	2097	1.2	1747	1.2	1.05 (0.59 - 1.86)	0.915
6 - 10 Yrs	3843	4.8	2278	3.3	1.47 (1.13 - 1.92)	0.003*
>10 Yrs	4318	7.5	5357	5.0	1.5 (1.29 - 1.76)	<0.001**
**Self-Identification**						
Predominantly Panthi (Anal-insertive)	1414	3.0	2847	3.7	0.81 (0.57 - 1.15)	0.227
Predominantly Kothi (Anal-receptive)	7965	5.7	3879	3.7	1.55 (1.29 - 1.86)	<0.001**
AC/DC or Double Decker (Anal- insertive and receptive)	2289	5.0	3246	4.2	1.19 (0.93 - 1.52)	0.158
Others	278	4.0	1153	1.6	2.40 (1.16 - 4.99)	0.016*
**Commercial Sex Behaviour**						
Yes	6870	5.0	6477	3.2	1.55 (1.31 - 1.84)	<0.001**
No	5076	5.5	4652	4.2		
**Alcohol consumed before or during sex^#^**						
Yes	3139	5.1	3528	3.3	1.55 (1.23 - 1.96)	<0.001**
No	2155	6.5	3038	2.8		
**Injected drugs Injected drugs^¥^**						
Yes	207	7.0	369	5.0	1.39 (0.72 - 2.71)	0.312
No	11744	5.1	10761	3.6		
**Any STI symptom present^$^**						
Yes	1924	5.7	2895	5.3	1.07 (0.85 - 1.36)	0.566
No	9998	5.1	8232	3.0		
**Heard of HIV/AIDS**						
Yes	11339	5.3	10678	3.7	1.43 (1.26 - 1.61)	<0.001**
No	599	3.9	451	1.9	2.04 (0.94 - 4.41)	0.084
**Ever tested for HIV**						
Yes	9011	5.7	8184	4.4	1.31 (1.15 - 1.49)	<0.001**
No	2312	3.5	2487	1.4	2.41 (1.63 - 3.57)	<0.001**

^ Others included: Skilled/Semi-skilled worker, Petty business/ Small shop, Large business/ self-employed, Service (private/government).

* Significantly differed at 5% level (P<0.05)

** Significantly differed at 0.1% level (P<0.001)

@ RR: Risk Ratio (Reference category Bisexual MSM); CI: Confidence Interval

# Among those who consumed alcohol

¥ Injected drugs for non-medical purposes (in last 12 months)

$ Had at least one STI symptom during the past 12 months (Vaginal discharge/lower abdominal pain without diarrhea or menses/Genital ulcer or sores)

STI - Sexually Transmitted infections

**Figure 1:**
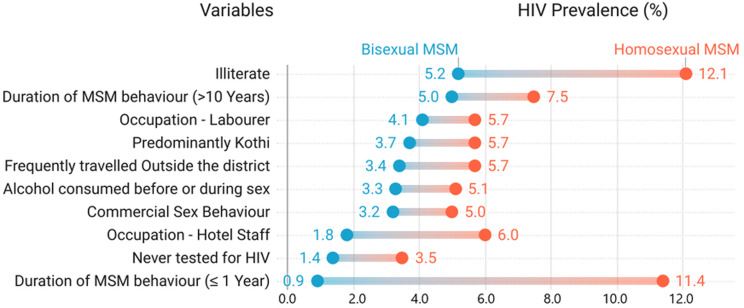
HIV Prevalence among Homosexual MSM and Bisexual MSM based on socio-demographic and behavioural variables

Certain factors contributed to a significantly higher risk of infection among homosexual MSM than the bisexuals, as represented in Figure 1. From the univariate analysis, with homosexual MSM, age, education, source of income, partner living with, traveling outside the district, duration of MSM behaviour, self-identified sexual orientation were significant factors independently associated with higher HIV prevalence. Within bisexual MSM, age, education, source of income, marital status, partner living with, age of initiation and duration of MSM behaviour, and presence of STI symptoms were independently associated with higher HIV prevalence. Based on the multivariable analyses, within homosexual MSM, HIV prevalence was significantly higher among illiterates, sex workers, those living with family without a sexual partner, those living with a male or hijra partner, those traveling outside their districts, new entrants (duration of MSM activity < one year) and those who identified themselves as kothi/panthi. Within bisexual MSM, HIV prevalence was significantly higher among older men, illiterates, those with no income (unemployed or student), sex workers, those living with family without a sexual partner, those living with a female partner, those who had their first intercourse with a male or hijra partner at age 24 or less, those with a duration of MSM activity of 6 or more years and those with any STI symptoms. (Table 2)

**Table 2:** Factors associated with HIV infection among Homosexual and Bisexual MSM by multivariable analysis

	Homosexual MSM			Bisexual MSM		

Factors	HIV (%)	OR (95% CI)	aOR (95% CI)	HIV (%)	OR (95% CI)	aOR (95% CI)

**Age Group (Yrs.)**						
15-24	3.7	Reference		2.3	Reference	
25-34	5.3	1.45 (1.20 - 1.75)**	1.12 (0.86 - 1.45)	3.0	1.35 (0.98 - 1.84)	0.89 (0.57 - 1.39)
≥ 35	8.8	2.48 (1.98 - 3.11)**	1.36 (0.95 - 1.95)	5.3	2.44 (1.81 - 3.29)**	1.84 (1.08 - 3.13)*
**Education**						
Literate	4.4	Reference		3.4	Reference	
Illiterate	12.1	3.03 (2.50 - 3.67)**	3.69 (2.9 - 4.69)**	5.2	1.56 (1.20 - 2.01)**	1.44 (1.07 - 1.95)*
**Source of Income**						
Unemployed/Student	4.0	0.92 (0.72 - 1.17)	1.19 (0.90 - 1.58)	3.9	1.40 (1.02 - 1.92)*	2.64 (1.82 - 3.83)**
Labourer	5.7	1.31 (1.05 - 1.64)*	1.16 (0.90 - 1.49)	4.1	1.48 (1.15 - 1.92)*	1.22 (0.92 - 1.63)
Domestic Servant	7.0	1.64 (0.94 - 2.85)	1.45 (0.77 - 2.72)	4.9	1.78 (1.03 - 3.07)*	1.74 (0.97 - 3.14)
Transport worker	1.6	0.35 (0.12 - 1.09)	0.45 (0.14 - 1.42)	2.1	0.74 (0.36 - 1.51)	0.63 (0.26 - 1.55)
Hotel Staff	6.0	1.38 (0.98 - 1.95)	1.41 (0.97 - 2.04)	1.8	0.62 (0.35 - 1.12)	0.57 (0.31 - 1.05)
Sex work/Masseur	9.9	2.41 (1.78 - 3.26)**	2.22 (1.55 - 3.16)**	8.3	3.12 (1.99 - 4.90)**	2.57 (1.46 - 4.52)**
Others^^^	4.4	Reference		2.8	Reference	
**Marital status**						
Never Married	5.2			3.3	Reference	
Currently Married	6.2			4.1	1.23 (1.00 - 1.52)	
Separated/Widowed/	3.6			1.5	0.43 (0.23 - 0.81)*	
Divorced/Others **Currently living**						
Living alone	3.5	Reference		2.4	Reference	
Family/relatives	5.5	1.60 (1.25 - 2.03)**	2.13 (1.6 - 2.82)**	3.7	1.60 (1.09 - 2.34)*	1.51 (0.99 - 2.29)*
without sexual partner						
Female partner	4.7	-		3.8	1.63 (1.11 - 2.39)*	1.52 (0.99 - 2.33)*
Male/Hijra partner	7.2	2.13 (1.35 - 3.38)**	1.70 (1.03 - 2.82)*	5.0	2.19 (1.03 - 4.65)*	0.55 (0.14 - 2.08)
Living with	5.0	1.43 (0.99 - 2.07)*	1.02 (0.63 - 1.66)	3.6	1.54 (0.86 - 2.76)	1.47 (0.76 - 2.86)
friends/others **Traveled Outside the District**						
No	4.8	Reference		4.0	Reference	
Yes	5.7	1.21 (1.03 - 1.42)*	1.35 (1.12 - 1.62)**	3.4	0.84 (0.69 - 1.03)	
**Age of first sexual intercourse with male/hijra (In years)**						

≤18	5.4	1.69 (0.84 -3.39)		4.3	3.96 (1.98 - 7.94)**	4.58 (2.21 - 9.5)**
19-24	5.8	1.83 (0.89 - 3.75)		3.3	3.03 (1.49 - 6.16)*	3.11 (1.51 - 6.4)*
≥25	3.3	Reference		1.1	Reference	
**Duration of MSM behavior (In years)**						
0 - 1	11.4	Reference		0.9	Reference	
>1-5	1.2	0.10 (0.06 - 0.16)**	0.09 (0.05 - 0.15)**	1.2	1.34 (0.39 - 4.56)	1.12 (0.32 - 3.91)
6 - 10	4.8	0.39 (0.27 - 0.57)**	0.32 (0.21 - 0.49)**	3.3	3.74 (1.16 - 12.07)*	3.64 (1.08 - 12.21)*
>10	7.5	0.62 (0.44 - 0.89)*	0.39 (0.24 - 0.62)**	5.0	5.79 (1.82 - 18.37)*	4.31 (1.23 - 15.13)*
**Self-Identification**						
Predominantly Panthi (Anal-insertive)	3.0	Reference		3.7	Reference	
Predominantly Kothi (Anal-receptive)	5.7	1.96 (1.42 - 2.7)**	1.5 (1.05 - 2.15)*	3.7	0.99 (0.77 - 1.28)	
AC/DC or Double Decker	5.0	1.72 (1.2 - 2.47)*	1.68 (1.13 - 2.5)*	4.2	1.15 (0.89 - 1.49)	
Others	4.0	1.34 (0.68 - 2.64)	0.4 (0.14 - 1.15)	1.6	0.44 (0.27 - 0.72)**	
**Commercial Sex Behaviour**	5.0	0.90 (0.76 - 1.06)		3.2	0.75 (0.62 - 0.92)*	0.63 (0.51 - 0.79)**
**Alcohol consumed before or during sex**	5.1	0.78 (0.61 - 0.98)		3.3	1.16 (0.87 - 1.54)	
**Injected drugs**	7.0	1.39 (0.81 - 2.38)		5.0	1.43 (0.89 - 2.31)	
**Any STI symptom present**	5.7	1.12 (0.91 - 1.39)		5.3	1.80 (1.46 - 2.21)**	2.02 (1.6 - 2.55)**
**Never Heard of HIV/AIDS**	3.9	0.73 (0.48 - 1.12)		1.9	0.51 (0.26 - 1.01)	
**Never tested for HIV**	3.5	0.59 (0.46 - 0.75)**	0.76 (0.58 - 0.99)*	1.4	0.32 (0.22 - 0.45)**	0.35 (0.24 - 0.51)**

aOR = Adjusted odds ratio; CI: Confidence Interval; *Significantly differed at 5% level (P<0.05); **Significantly differed at 0.1% level (P<0.001)						

### Socio-demographic Factors

Among bisexual MSM, HIV prevalence was significantly higher among older men aged 35 years and more (5.3%; aOR:2.44; CI:1.81 - 3.29; P<0.001). Further, the odds of infection were significantly higher when the bisexual MSM had their first intercourse with a male or hijra at a young age (< 24 years). Although the trends were similar among homosexual MSM, these factors were not significantly associated with HIV prevalence after adjusting for the confounding factors. Although almost 90% of the MSM were literates, HIV prevalence was significantly higher among the illiterates in both homosexual (12.1%; aOR:3.69; CI:2.9-4.69; P<0.001) and bisexual (5.2%; aOR:1.44; CI:1.07-1.95; P<0.05) groups. Based on the source of income, HIV prevalence was significantly higher among homosexual MSM who were masseurs or sex workers (9.9%; aOR:2.22; CI:1.55-3.16; P<0.001). In the bisexual group, HIV prevalence was significantly higher among unemployed/students and masseurs or sex workers (8.3%; aOR: 2.57; CI:1.46-4.52; P<0.001). HIV prevalence was higher among the currently married MSM in both homosexual and bisexual groups when compared to those who were never married or separated/widowed (Table 2). In both groups, the prevalence was significantly higher among MSM living with family without a sexual partner when compared to those living alone. Likewise, HIV prevalence was significantly higher among those living with a male or hijra partner in the homosexual group (7.2%; aOR: 1.70; CI:1.03-2.82; P<0.05) and those living with a female partner in the bisexual group (3.8%; aOR: 1.52; CI: 0.99-2.33; P<0.05) (Table 2). Traveling outside the residing district was significantly associated with HIV prevalence among homosexual MSM. While the risk of infection was significantly higher among the new MSM (Duration of MSM activity = 0-1 year) in the homosexual group, it was higher among those with a longer duration of MSM activity (> 5 years) in the bisexual group.

### Behavioural Factors

**Figure 2:**
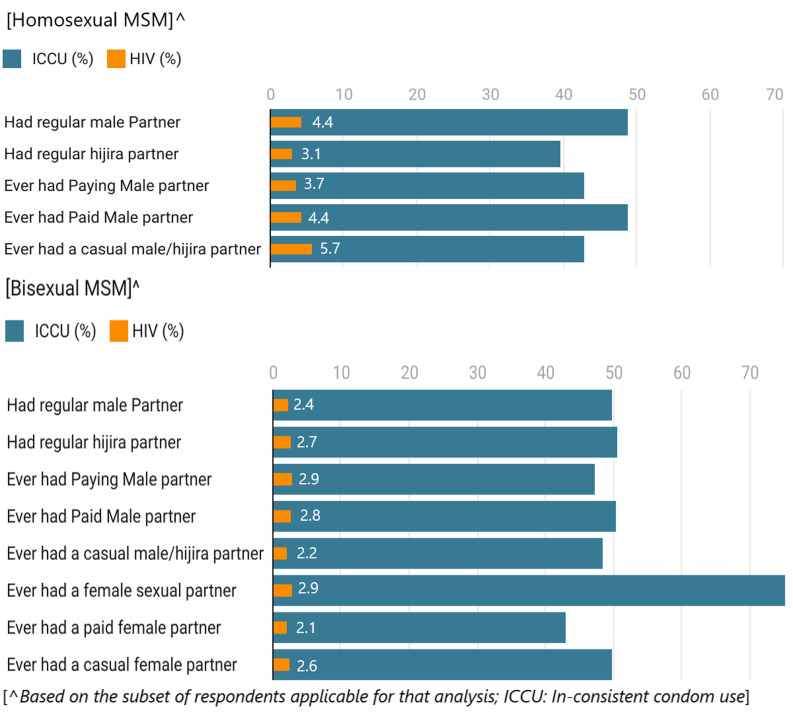
Inconsistent Condom usage and associated HIV prevalence among Homosexual and Bisexual MSM based on partner types

About half the MSM in both groups had a regular male partner and paying male partners. The proportion of those having hijra partners or those who ever paid a male partner was comparatively lesser. Likewise, nearly half (48.2%) of the bisexual MSM had female partners; those with casual female partners were comparatively lesser (18.5%). Only about half the MSM in both groups were consistent in condom usage, which varied between partner types (Table 3). The proportion reporting inconsistent condom usage was highest (75.3 %) with female partners among bisexual MSM and the lowest (39.7 %) with hijra partners among homosexual MSM (Figure 2). The prevalence was comparatively higher among those reporting consistent condom usage in both groups. Based on sexual partner types and consistency in condom usage, HIV prevalence ranged from 3.1% to 8.5% among homosexual MSM and 2.1% to 5.1% among bisexual MSM (Table 3).

**Table 3:** Condom usage and associated HIV prevalence among Homosexual and Bisexual MSM based on partner types

Partner Type	Homosexual MSM (N =11951)			Bisexual MSM (N= 11130)			Overall	
	%^	CCU % (HIV %)	ICCU % (HIV %)	%^	CCU % (HIV %)	ICCU % (HIV %)	(%)	HIV %
Had regular male Partner	53.7	51.1 (6.2)	48.9 (4.4)	55.4	50.2 (4.4)	49.8 (2.4)	54.5	4.2
Had regular hijira partner	17.1	60.3 (4.1)	39.7 (3.1)	26.7	49.3 (4.7)	50.7 (2.7)	21.7	3.5
Ever had paying Male partner	50.7	57.0 (5.6)	43.0 (3.7)	45.9	52.6 (4.9)	47.4 (2.9)	48.4	4.3
Ever Paid a Male partner	21.4	51.1 (8.5)	48.9 (4.4)	31.8	49.6 (4.7)	50.4 (2.8)	26.4	4.4
Ever had a casual male/hijira partner	37.4	57.0 (3.2)	43.0 (5.7)	36.8	51.5 (3.7)	48.5 (2.2)	37.1	3.9
Ever had a female sexual partner		-	-	48.2	24.7 (5.1)	75.3 (2.9)	48.2	3.6
Ever had a paid female partner		-	-	24.5	56.9 (3.3)	43.1 (2.1)	24.5	2.4
Ever had a casual female partner		-	-	18.5	50.1 (4.2)	49.9 (2.6)	18.5	3.5

CCU: Consistent condom use; ICCU: In-consistent condom use; “Consistent condom” use was defined as using a condom at each sex act (every time) with a partner in the last month.

^ Among those how had a partner

Data suggests that the proportion of MSM with commercial sex behaviour and alcohol consumption during sex did not vary much between the groups. However, the proportion of those injecting drugs (3.3% vs. 1.7%) and having STI symptoms (26% vs. 16.1%) was comparatively higher in bisexual MSM than homosexual MSM. HIV prevalence was higher among those injecting drugs and having STI symptoms in both groups. Having STI symptoms was significantly associated with HIV prevalence among bisexual MSM (5.3%; aOR:2.02; CI:1.6 - 2.55; P<0.001).

## Discussion

A noticeable decline in HIV prevalence among heterosexual populations in India has been evident in recent years; however, HIV prevalence among MSM is much higher than the adult HIV prevalence. Homosexuality was not legally recognized in India until 2018, and MSM had been one of the coverts and marginalized HRGs. The legalization of section 377 in 2018 might be a preliminary step towards self-declaration of homosexuality and can gradually change the general public's perceptions of sexual orientation.^13^ It is, therefore, anticipated to be a supportive change for effective HIV interventions among MSM. While HIV risk is highly confined among MSM, it is highly probable for an MSM to be in a heterosexual marriage to satisfy the socio-cultural norms of India or due to stigma or fear of rejection. Reports show that most MSM do not disclose their homosexuality to their family or partners.^14^ Bisexual MSM act as bridge population and can transmit the infection to their female partners. Periodic analysis of socio-demographic and behavioural patterns of homosexual and bisexual MSM will be instrumental in identifying the dynamics of transmission risks to channelize the targeted interventions.

HIV prevalence among homosexual MSM was significantly higher than that of bisexual MSM. As reported in most studies worldwide, the univariate analysis shows an age-dependent increase in HIV prevalence in both groups. The higher prevalence among older MSM is attributed to increased exposure, changing behaviours with age, unprotected anal intercourse, and sexual behaviors.^6^,^15^-^17^ The studies also claim that lower prevalence among younger HRGs could be due to their increased knowledge in HIV prevention and their ability to negotiate safe behaviors.^15^ The proportion of older men was higher among bisexuals (33.1% vs. 14.1%). Previous studies highlight the significant association of older age with bisexual behaviour and report subjective evidence of changing sexual identities to bisexuality among older MSM.^6^ Reports also suggest the possibility of reduced testing and diagnosis among older men, increasing their infection risks.^18^ Further studies focusing on testing status and changing sexual behaviours with older age among Indian MSM will ensure the effectiveness of the MSM interventions.

Lower levels of education and lesser or no income are often associated with higher HIV prevalence among MSM. The univariate analysis results indicate a higher prevalence among illiterates than literates, laborers, domestic servants, unemployed or students, hotel staff, and male sex workers/masseurs. While less than 6% of MSM in both groups were MSW or masseurs, nearly 50% reported having commercial sex and had paying partners, indicating that most MSM sort to commercial sex for monetary benefits, making them vulnerable to unsafe sexual behaviours.

Younger age at initiation of intercourse with a male or hijra partner was significantly associated with higher prevalence. Younger MSM are susceptible to HIV infection due to their anatomical and physiological conditions.^19^ Several factors, including abused childhood, peer pressure, psychological factors, social media, and curiosity, influence early sex, leading to unsafe behaviours. It is also reported that unsafe sexual behaviours are more likely in young men than women.^20^,^21^ Almost 70% of the MSM in this study had their first homosexual encounter within 18 years of age, of which over 25% reported forced homosexuality. The findings align with a mixed-methods, multi-site, Indian study that reported a high prevalence of child sexual abuse (CSA) among MSM (22.4%) and its significant association with HIV-related risk factors.^22^ Child sexual abuse (CSA), especially among boys in India, is often less spoken of, and hence the CSA-related interventions are essential in the HIV prevention services.

MSM with a duration of MSM activity lesser than one year are considered new entrants and are most vulnerable to unsafe behaviours. In our study, the proportion of inconsistent condom usage with any partner type was higher among new entrant homosexual MSM, which may explain the higher prevalence among new entrant homosexual MSM. On the contrary, the proportion of bisexual MSM exhibiting inconsistent condom usage with regular female partners and needle sharing behaviour increased with the duration of MSM activity, increasing their infection risks. Rejection among peers, inability to negotiate safe sex, long-term exposure, and the reluctance of married or bisexual MSM to seek HIV services are reported to be few reasons for higher infection risks among bisexuals or married MSM with longer duration of MSM activity.^23^ A higher proportion of MSM who self-identified as double-deckers or bisexuals/others in both groups reported inconsistent condom usage than those identified as either predominantly kothi or predominantly panthi.

Living with a partner or friends was significantly associated with a higher infection risk than living alone. Among homosexual MSM, those living with a male/hijra partner had a significantly higher infection, and inconsistent condom usage was higher among regular male or hijra partners. Inconsistent condom usage with regular partners is often attributed to status disclosure, trust, need for intimacy, and power differentials in relationships.^24^ The regular female partners of bisexual MSM are most likely to be their wives/girlfriends, and bisexual MSM hide their homosexual identities to their female partners, fearing rejection. Previous studies have reported bisexual MSM bridging HIV transmission between high-risk and general populations. Accordingly, inconsistent condom usage was the highest (75%) with regular female partners, the inconsistency increasing with the duration of MSM behaviour. Condom-less behaviour or inconsistent condom usage with regular female partners has been persistently highlighted in most studies.^23^,^25^ A qualitative study reports that consistent condom usage with other men, reluctance to use with wives, regular HIV testing in case of exposure risks, desire to have children and fear of discriminations or accusations of unfaithfulness were some of the reasons stated by married MSM for not using condoms with their wives. For wives of MSM, condoms were more often considered for birth control. The men were the primary decision-makers of condom usage, to which they complied even if they were aware of their partners' homosexual behavior.^25^ Thus, prevention services that imply safe sex practices considering the relationship characteristics of the MSM might be more insightful to prevent heterosexual transmission. In an era of multiple prevention products other than condoms, other safe practices such as pre-and post-exposure prophylaxis (PrEP, PEP), ART adherence, and safe injecting practices that reduce the transmission risk of regular partners are to be encouraged. A recent analysis among MSM in France reported an increased uptake of PrEP and decreased condom usage with an overall increase in the rate of protected anal sex.^26^

Substance abuse is often linked to unsafe behaviours, which in turn increases the infection risk.^27^-^29^ Previous studies among Indian MSM report the association of alcohol consumption to increased odds of inconsistent condom^30^ and injecting drug use (IDU) with higher HIV prevalence among MSM.^31^ In our analysis, more than 50% of MSM in both groups reported alcohol consumption during or before sex, but the behaviour was not associated with higher HIV prevalence. On the other hand, the proportion of MSM with IDU practice (MSM-IDU) was below 5% in both groups. However, the HIV prevalence among MSM-IDU was significantly higher in both groups when compared to those who do not inject drugs. Specifically, the proportion of bisexual MSM with IDU was comparatively higher than the homosexual MSM. Studies document higher levels of psychosocial issues among bisexual MSM, which were often associated with high-risk behaviours such as substance abuse, unsafe sexual behaviours, and multiple sexual partners.^6^,^32^ Accordingly, the proportion reporting inconsistent condom usage was considerably higher with all partner type other than regular female partners among bisexual MSM with IDU behaviour (59.2% to 68.7%) than the bisexual MSM without any IDU behaviour (40.4% to 49.3%). Needle sharing was more prevalent among illiterates (63.8%) and sex workers (45.8%) among homosexual MSM with IDU behaviour than their counterparts. Nevertheless, IDU practice among MSM was geographically confined, so localized integrated-targeted interventions at such regions may be beneficial.

## Conclusion

This study provides preliminary evidence of factors associated with higher HIV prevalence among homosexual and bisexual MSM in India. Older age, low education, being a sex worker, being married, living with a male or hijra partner, younger age at initiation of MSM behaviour, injecting drug use, and having STI symptoms were associated with higher HIV prevalence in both groups. Further, among homosexual MSM being new to MSM was a significant risk factor with a prevalence of 11.4%. Measures for early identification and dissemination of the HIV prevention services to the new entrant MSM must be strengthened. Bisexual MSM poses a severe risk of heterosexual transmission, with nearly 75% of the bisexual MSM reporting inconsistent condom usage with female partners. Safe sex practices must be advocated to curb bridge transmission. Progressing towards End of AIDS, a deeper understanding of the underlying risk factors and stratified, novel interventions are essential to optimize the targeted interventions.
